# Everybody's Page

**Published:** 1890-07-05

**Authors:** 


### Everybody's Page.

While in England and Belgium solitary confinement is endured well by prisoners, in France the effect is often mental derangement or suicidal tendency.

---

Fishing by torch-light seems a curious occupation. Yet, such are the means by which the fishermen of Sainte Croix and Puerto in the Canary Isles carry on their industry. The waters round these islands abound in every variety of fish.

---

The British Consul at Santos appears to have a great belief in the future of Brazil. Capital and immigration, he states, are alone wanting in order to give this province a start. He appeals to Englishmen to be first in the field, but Englishmen who have already invested many millions in the country probably will like to see how the new government gets on first.

---

A young man in Vienna was evidently desirous of emulating the fate of the gentleman in history who was drowned in a butt of Malmsey wine. He has drowned himself in a barrel of vinegar which he decorated with three crosses and his initials before he got into it. We feel he must either have fancied himself a martyr or else have been ignorant of how painful it is to swallow even a small quantity of vinegar.

---

Enquiring Patient:

"Can you explain to me,
Why all physicians take
A guinea for their fee
When we no guineas make?

Loyal Doctor replies:

"Oh, yes, the reason's plain,
They are loyal and unwilling
That a sovereign e'er again
Should be left without a shilling."

---

"Grub is cheap to-day. Only 3d. a time!" How appetising this advertisement, which appears in a window in a well-known London thoroughfare, must look to the hungry and footsore knights of the pavement. The mental arithmetic through which they go in calculating how much they can stow away for 3d., and for how many days they can make the meal last, must sharpen their appetites considerably. But the pang must be bitter when they find, as we did, on closer inspection, that the 3d. grub is a mental one, only consisting of old books provided for the delectation of the bookworm!

---

According to Dr. Dymock, of Bombay, the betel-nut, which is consumed very largely in India, has, when fresh, intoxicating properties, and produces giddiness. Heat diminishes the intoxicating power of the nut. It would be rather awkward if areca-nut tooth-paste had the property of intoxication as well as astringency.

---

Sweet potatoes, which are consumed on a large scale in the British West Indies, can be dried as easily as other fruits by slicing and exhausting them of their moisture, which does not detract in any way from their nutritious properties. As the plant can be grown so easily, the export of dried sweet potatoes should be an extensive and profitable industry.

---

The statement that during the cold weather the temperature at night at the hill stations in Upper India, the average elevation of which may be taken as 7,000 ft., is often higher than at the plain stations at an average height of 1,000 ft., seems a curious inversion of the normal state of affairs. This is the case especially in fine clear weather, when there is little horizontal movement of the air. The temperature above the plains is then determined by that of rapidly ascending moist and unsaturated air rising with the maximum temperature at the level of the plains. As a steady flow takes place from the hills at night down to the plains another flow of comparatively warm air travels towards the hills.

---

We notice that the fifteenth annual meeting of the Sunday Society was held recently, and yet we have not achieved the opening of our museums and picture galleries on Sundays. The three considerations against Sunday opening are, firstly, those founded on religious motives—"the desecration of the Lord's Day"; secondly, there is a feeling that public employés would be deprived of their day of rest; and, thirdly, that a great number of the working classes are absolutely indifferent to the matter. Speaking in all toleration, these objections present to us no difficulty. If the working classes are indifferent, and we doubt it very much, it is only lack of education which makes them so; therefore, they more than anybody else need extra facility for acquiring knowledge. The Sunday officials need not be those of the weekdays, neither need the same officials attend every Sunday. And, lastly, how is Sunday desecrated by our spending our afternoons in studying and enjoying the beauties and wonders of the world?

